# Computed tomography texture analysis to discriminate fungal and non-fungal infected fluid collections

**DOI:** 10.1117/1.JMI.10.6.064002

**Published:** 2023-11-09

**Authors:** Benedikt Schnarkowski, Jakob Leonhardi, Matthias Mehdorn, Ines Gockel, Gordian Prasse, Sebastian Ebel, Timm Denecke, Hans-Jonas Meyer

**Affiliations:** a University of Leipzig, Department of Diagnostic and Interventional Radiology, Leipzig, Germany; b University of Leipzig, Department of Visceral, Transplant, Thoracic and Vascular Surgery, Leipzig, Germany

**Keywords:** texture analysis, computed tomography, fungal infection, fluid collection

## Abstract

**Purpose:**

Texture analysis of computed tomography (CT) can aid in characterization of fluid collections providing biomarkers. The present study tested whether texture analysis can discriminate between fungal or non-fungal infection in patients undergoing CT-guided percutaneous drainage treatment.

**Approach:**

Overall, 214 patients [(n=76 females, 35.5%); mean age 62±14 years and range 20 to 94 years] with 255 fluid collections were included in the analysis. All patients underwent CT-guided drainage treatment and were evaluated with microbiological analysis. CT texture analysis was performed with the MaZda package.

**Results:**

Only three of the investigated CT texture features were statistically significant different between the groups, namely kurtosis (p=0.04), S(3,3)InvDfMom (p=0.02), and S(5,-5)DifEntrp (p=0.003). These texture features were further investigated by the receiver operating characteristic curve. S(3,3)InvDfMom achieved the highest accuracy with an area under the curve of 0.62, resulting in a sensitivity of 0.66 and a specificity of 0.57.

**Conclusion:**

Some CT texture features were different between fungal and non-fungal infected fluid collections. The diagnostic overlap is large, which could reduce the clinical benefit. Further studies are needed to identify the possible diagnostic benefit of texture analysis in these patients.

## 1 Introduction

Fluid collections occur commonly and can represent several entities comprising seroma, biloma, hematoma, ascites, lymphocele, and abscess, which depend on the localization and cause.^1^^–^^4^ Depending of the entity of the fluid collection, immediate treatment can be of utmost importance for the patient.^4^^–^^10^

Computed tomography (CT) is the diagnostic modality of choice in patients with fluid collections to diagnose septic foci and to evaluate the treatment, including the possible percutaneous access.

A common treatment option is CT-guided percutaneous drainage, whereas surgery is only performed in few selected cases nowadays, irresponsive to radiologic-interventional evacuation.^5^^,^^8^^,^^9^

Some CT imaging features have been proposed to be indicative of an infection of a fluid collection, which comprise contrast media enhancement with rim-like appearance, air entrapment, and higher Hounsfield unit (HU) attenuation. However, single imaging features by itself yield not enough specificity and therefore cannot reliably exclude infection.^3^^,^^4^^,^^11^

Texture analysis is a modern quantitative imaging analysis based on radiological images to provide novel imaging biomarkers.^12^^–^^14^ Most commonly, CT images are employed due to its robust imaging acquisition and high availability in clinical routine.^12^^–^^14^

In the literature, texture analysis was predominantly investigated in the field of oncological imaging to better characterize tumor behavior and to provide novel applications of imaging to guide oncological patients.^12^ Beyond that, CT texture features can provide information regarding the microstructure of tissues, which was shown in preliminary studies.^13^^–^^19^ This is why texture features might also help to better characterize the content and different aspects of fluid collections to identify fungal associated changes, such as hyphae.

To date, the exact diagnosis of underlying causes pathogens of an infected fluid collection is beyond the scope of imaging and can be provided by microbiological analysis only.^20^ That is the reason why few systematic data exist to discriminate fungal and bacterial infected fluid collections based upon imaging.^20^ In clinical routine, it could be of interest to predict the underlying cause, as treatment options differ significantly between fungal and non-fungal fluid collections. Moreover, it could enable a new method to use the radiological images.

Therefore, the present study aimed to elucidate the differences between fungal and non-fungal infected of fluid collections employing CT texture features.

## 2 Methods

All patients undergoing CT-guided percutaneous drainage treatment were retrospectively screened in the radiological database of our university hospital between January 2017 and December 2020.

The inclusion criteria of the study:

- contrast enhanced CT for septic evaluation within 24 h before the drainage intervention,
- sufficient native planning CT of the intervention, and
- available clinical chemistry and microbiological data.

Finally, 214 patients (n=76 females, 35.5%), mean age 62±14 years, range 20 to 94 years) with 255 overall fluid collections, were included into the present analysis with sufficient imaging and clinical data. The patient sample was previously investigated to evaluate CT texture features for the discrimination between sterile and infected fluid collections.^21^

### 2.1 Clinical Data Acquisition

The clinical medical records were retrospectively analyzed to extract the clinical data: age, sex, medication with immunosuppressive drugs, current treatment with antibiotics, underlying disease, previous surgery, body temperature and serological parameters comprising C-reactive protein, white blood cell count, and procalcitonin and interleukin-6 within 6 h before the CT-guided intervention.

### 2.2 Image Acquisition

In all patients, CT imaging was performed in clinical setting with a 128 or 256 slices CT scanner (Ingenuity, Philips, Amsterdam, Netherlands/iCT, Philips, Amsterdam, Netherlands). 90 mL of contrast medium was given at a rate of 1.5 to 3.5  mL/s by a power injector (Accutron CT, Medtron GmbH, Germany) with a scan delay of 70 s after onset of injection for clinical evaluation of septic foci. Typical CT parameters were 150 to 300  mAs/120  kVp. The images were reconstructed in three dimensional planes with a slice thickness of 1 mm.

Then, for all patients, a CT-guided intervention was performed. The CT-guided drainage therapy was performed with a 16 slices CT scanner (Big Bore 16, Philips, Amsterdam, Netherlands). For all patients, a native CT spiral scan was performed for treatment planning and to plan the percutaneous access to the fluid collection. CT imaging parameters were 120 kVp, 150 mAs with a slice thickness of 2 mm.

In all cases, a percutaneous drainage was safely placed within the fluid collection. The size of the drainage ranged from 8 to 16 French, which depended on clinical presenting and localization of the fluid collection.

### 2.3 Texture Analysis

**Fig. 1:**
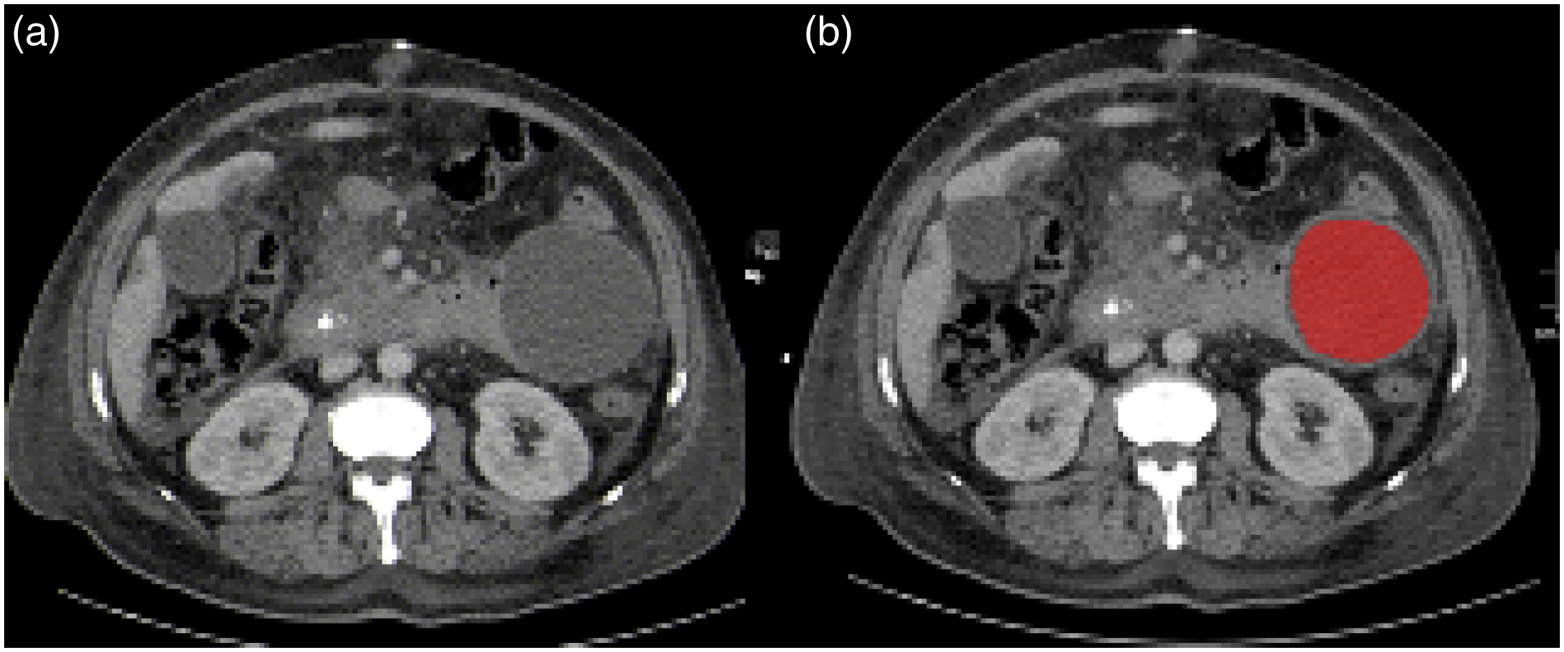
Representative case of the patient sample with a large fluid collection peritoneally on the left side caused by a fungal infection. (a) The portalvenous phase image with the fluid collection. (b) The ROI is highlighted in red color on the representative slice of the fluid collection.

The CT images were extracted and further analyzed with the free available software MaZda (version 4.7, available at Ref. 22).^23^^,^^24^ Only the contrast enhanced CT images in portal-venous phase were used for texture analysis. This was used to reduce heterogeneity of the texture features. The measurements were performed on the largest, representative slide within the boundary of the fluid collection using a polygonal region of interest (ROI). Gas bubbles were excluded of the ROI to reduce possible heterogeneities of the texture features. The radiologist was blinded to the microbiology results. Gray-level normalization was utilized to minimize the influence of contrast and brightness variation, as it is commonly employed in similar studies employing the limitation of dynamics to μ±3 SD (μ gray level mean, SD standard deviation).^25^ The extracted features include several first and second order texture features: gray-level histogram, co-occurrence matrix, run-length matrix, absolute gradient, autoregressive model, and wavelet. For every fluid collection, 279 texture features were quantified. Figure 1 displays a representative case of the patient sample with a fungal infected fluid collection for illustrative purposes.

### 2.4 Microbiological Analysis

Immediately after CT-guided puncture of the fluid collection, at least 5 mL of the drained fluid were transported at room temperature to the microbiology department for subsequent microbiological analyses. For all cases, the same laboratory was used for the analysis. Microscopic inspection and Gram staining were performed to analyze the samples of the fluid collections. Agar plates were also employed as a culture medium for microorganisms. If microscopic and Gram stain results, as well as cultures, were negative, infection was deemed to be non-existent.

If leukocytes and bacteria were discovered on the Gram stain and/or the culture was positive, the fluid was declared infected by current clinical standards. The investigated patient groups were divided into fungal and non-fungal cause accordingly. The exact bacteria and fungi were extracted.

### 2.5 Statistical Analysis

Statistical analysis was performed using GraphPad Prism 5 (GraphPad Software, La Jolla, CA, USA) and SPSS STATISTICS (IBM, Version 25.0; Armonk, New York, United States). Collected data were evaluated by means of descriptive statistics. Possible differences of the texture features between fungal-infected and non-fungal infected fluid collections were investigated by the Mann–Whitney test or Fisher’s exact test, when suitable. Diagnostic accuracy was tested with receiver operating characteristics (ROCs) analysis. In all instances, p-values<0.05 were used to indicate statistical significance.

## 3 Results

### 3.1 Clinical and Radiological Parameters

Table 1 provides the descriptive statistics of the investigated demographic, clinical, serologic, and CT imaging parameters.

**Table 1:** Overview of the investigated clinical and imaging features.

Parameter	Fungal-infected (n=41)	Non-fungal infected (n=214)	p-value
Age, years (range)	58 ± 14 (23 to 81)	63 ± 14 (20 to 90)	0.03
Sex, male/female	24/11	114/65	0.7
Diabetes	8 (22.9%)	54 (30.2%)	0.5
Immunosuppresive drugs	0 (0%)	31 (17.3%)	0.003
Antibiotics	26 (74.3%)	146 (81.6%)	0.4
C-reactive protein, mg/l	293 ± 89 (68 to 429)	182 ± 103 (1 to 435)	0.2
Leukocytes, 10*9/l	16.9 ± 7.2 (7 to 32.8)	15± 7.8 (2.3 to 81.2)	0.09
Procalcitonin, ng/ml	5.2 ± 11.7 (0.1 to 47.9)	6.2 ± 16.6 (0.1 to 108.5)	0.6
Interleukin-6, pg/ml	2686 ± 3700 (59 to 6918)	3141 ± 7573 (38 to 28687)	0.6
Attenuation, HU	18 ± 6 (6 to 33)	19 ± 9 (0 to 58)	0.6
Maximum diameter, cm	8.6 ± 3.6 (2.1 to 15.4)	8.2 ± 3.3 (2.6 to 20)	0.3
Wall enhancement	24 (56%)	129 (60.3%)	0.9
Fat stranding	22 (54%)	139 (64.9%)	0.2
Entrapped gas	31 (76%)	117 (54.7%)	0.02
Kurtosis	0.2 ± 0.2 (−0.3 to 0.7)	0.1 ± 0.2 (−0.4 to 0.9)	0.04
S(3,3)InvDfMom	0.1 ± 0.01 (0.08 to 0.14)	0.1 ± 0.01 (0.07 to 0.15)	0.02
S(5,-5)DifEntrp	1.4 ± 0.5 (1 to 2)	1.2 ± 0.4 (1 to 2)	0.03

Note: Data are expressed as mean ± standard deviation (range) or frequencies with percentages. P-values are calculated with Mann–Whitney test or Fisher’s exact test where appropriate.

Most patients had one fluid collection. In 37 cases (17.2%), 2 fluid collections were identified, and in 2 cases (1.4%), 3 fluid collections were identified. In patients with more than one fluid collection, all of them were punctured with independent microbiological work up.

There were 214 non-fungal infected (83.9%) and 41 fungal infected fluid collections (16.1%).

The most common identified bacteria were E. coli n=83 (38.8%), *Enterococcus* spp. n=75 (35%), *Streptococcus* spp. n=35 (16.4%), *Klebsiella* spp. n=29 (13.6%), *Bacteroides* spp. n=20 (9.3%), and *Staphylococcus* spp. n=19 (8.9%).

The most common fungal were *Candida albicans* infections with n=31 (75.6%), *Candida glabrata* with n=13 (31.7%), and *Candida krusei* with n=3 (7.3%).

### 3.2 Diagnostic Accuracy of the Investigated Texture Features

Regarding the clinical parameters, age and usage of immunosuppressive drugs were statistically significant different between patients with fungal- and non-fungal infection (p= 0.03 and p=0.003, respectively) (Table 1). For conventional imaging findings, only entrapped gas was different between the groups (p=0.02).

Only three of the investigated CT texture features were statistically significant different between the groups, namely kurtosis (p=0.04), S(3,3)InvDfMom (p=0.02), and S(5,-5)DifEntrp (p=0.003).

**Fig. 2:**
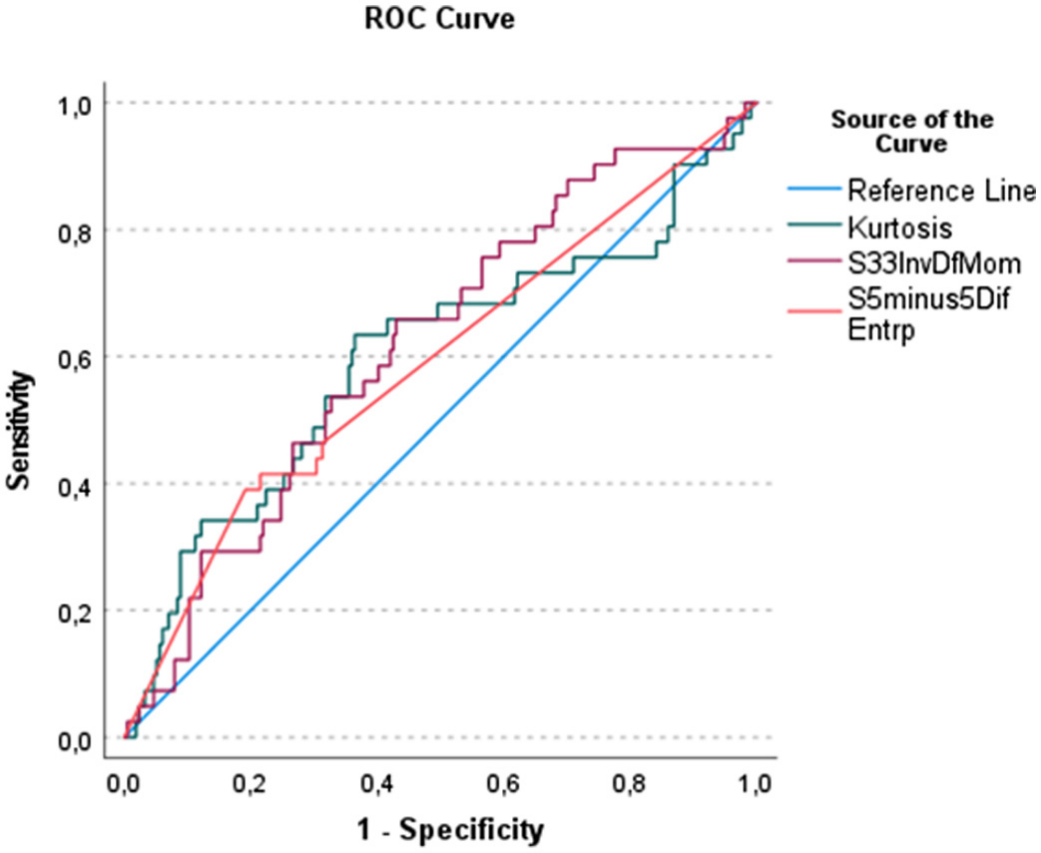
ROC-graph of the statistically significant texture features between fungal and non-fungal infection. The best AUC achieved “S(3,3)InvDfMom” with 0.62 resulting in a sensitivity of 0.63 and a specificity of 0.64.

These texture features were further investigated by ROC analysis (Table 2). S(3,3)InvDfMom achieved the highest accuracy with an AUC of 0.62, resulting in a sensitivity of 0.66 and a specificity of 0.57 (Fig. 2).

**Table 2:** Diagnostic accuracy of the CT texture features to discriminate fungal and non-fungal infected fluid collections.

CT texture feature	AUC	95% CI	Sensitivity	Specificity
**Kurtosis**	0.59	0.49; 0.70	0.63	0.64
**S33InvDfMom**	0.62	0.52; 0.71	0.66	0.57
**S5minus5DifEntrp**	0.59	0.49; 0.69	0.39	0.81

Note: AUC = area under the curve; CI = confidence interval.

## 4 Discussion

The present study clearly elucidated, whether CT texture analysis can predict the infectious etiology of a fluid collection reliably. This could aid in clinical decision-making, as imaging is performed in clinical routine for treatment planning. In this regard, the antibiotics treatment could be altered, when CT texture analysis is indicating a fungal infection before the microbiological confirmation is provided. In short, this could lead to an earlier treatment of fungal infected fluid collections.

Some CT signs were described to be indicative of an infection of a fluid collection comprising presence of gas entrapment, increased attenuation, encapsulation with or without wall enhancement and fat stranding of the surrounding tissue.^3^^–^^6^^,^^26^ It is a known fact that these imaging findings separately considered cannot reliably predict or exclude an infection of a fluid collection. This was exemplarily shown in a study with 92 patients, reporting a sensitivity of 83.4% and a specificity of 39.3% using the CT signs gas entrapment and high attenuation (20 or greater HU).^3^

Only a few studies tried to discriminate fungal from bacterial fluid collections as it was not within the scope of CT imaging to provide the underlying cause of inflammation. Hypothetically, bacterial infectious foci should form different microstructural inflammatory tissues compared to fungi.^27^ Notably, even on the microscopic level, some bacteria can mimic fungal infection.^28^ Another important point is the formation of mycelium and hyphae, which should result in a different microstructure compared to non-infected and bacterial infection.

A main rationale of the present analysis is that texture features could better characterize fluid collections with the ability to quantify heterogeneities of radiological images.^12^ Presumably, different infectious foci should lead to a slightly different CT image, which cannot be diagnosed by the radiologist. This was shown in previous studies in the oncologic field, who could employ texture analysis to reflect histopathologic features of tumors.^15^^–^^17^ It seems plausible to translate these results to non-oncological disorders.

It is widely acknowledged that higher attenuation of a fluid collection reflects inflammatory debris.^2^^,^^3^ Haemorrhage can also be the cause of higher attenuation in non-infected or infected fluid collections. Higher CT density over the threshold of 10 HU was the strongest predictor of infected fluid collections, with a good diagnostic accuracy, which was shown in a recent study.^21^

Texture analysis of fluid collections has only been performed in few previous studies.^20^^,^^21^^,^^29^ In a previous study on 320 patients, texture features were not diagnostic superior to conventional imaging findings to discriminate infected from sterile fluid collections.^20^ However, in this study, bacterial and fungi were not further investigated by subanalysis, which could result in these negative results. Based upon this study, it was further deliberated that different microbiological pathogens might result in different CT texture features. This could lead to a high heterogeneity in the mentioned study to further adjust for the underlying cause of the fluid collection.

In another study collective from Romania, 82 patients with gastric cancer undergoing surgery were investigated.^29^ The authors of this study could identify 10 texture features, which were statistically significant different between sterile and infected fluid collections, which highlights the possible use of texture analysis but is in distinctive contrast to the other above mentioned study.

Finally, by the same work group, “ATeta3” and “ATeta4” were highlighted as possible texture features to discriminate bacterial from fungi infected fluid collections based upon 42 patients.^20^

The three identified texture features of the present study were of different groups. The feature kurtosis is a first order histogram parameter, which reflects the slope of the histogram of the HUs.^12^^,^^14^ The other two parameters, namely S(3,3)InvDfMom and S(5,-5)DifEntrp are second order texture features, which reflect the heterogeneity of the voxels in a higher order and their relationship to each other in a spatial dimension.^12^

A recent study based on 58 patients investigated the diagnostic value of dual-energy spectral CT.^30^ The authors could identify that monoenergetic images could improve the diagnostic accuracy and showed a higher signal to noise ratio.^30^ The conclusions drawn of this study were that spectral reconstructions could improve the assessment of infected fluid collections and the help to depict the wall enhancement. However, the quantitative measurements of the spectral reconstructions were not superior compared with measurements in conventional images^30^ There is definite need to further evaluate the possible benefit of texture analysis derived from dual-energy CT. Other authors elucidated the diagnostic benefit of HUs or clinical scores with mixed results.^31^^,^^32^

The present study corroborates the abovementioned findings that texture analysis might aid to diagnose the etiology of fluid collections in a non-invasive and time efficient manner. However, the diagnostic overlap identified in the present study is relative large, which could reduce the benefit for clinical routine.

Contrary to the two previous studies of Romania, the present analysis included pleural fluid collections suspicious for empyema and fluid collections in non-surgical patients. This leads to larger patient spectrum, which could also lead to certain heterogeneities. However, the present results can be considered as representative for all fluid collections undergoing CT-guided percutaneous drainage with resulting larger external validity.

Despite the statistically significance of the three identified CT texture features, the clinical significance remains still unknown. The identified AUC is rather low, and the CT texture features on their own might be not be able to reliably predict the infectious status of the fluid collections.

More data are clearly needed to further elucidate the benefit of CT texture analysis in fluid collections.

The present study is not free of limitations. First, it is a retrospective study with inherent known bias. Yet, imaging and microbiology analyses were performed blinded and independently to each other to reduce possible bias. Second, possible selection bias has to be acknowledged, as only treated fluid collections were included. Some small fluid collections might not be treated by this procedure, which could show other associations with texture features. Third, the sample size is rather small and heterogeneous, which could lead to certain bias. A multicentric analysis is needed to further evaluate the diagnostic benefit of CT texture analysis in patients with fluid collections. Fourth, CT texture features are influenced by several technical factors, including contrast media phase, reconstruction algorithm, and CT scanner, which still needs to be addressed.^33^ Moreover, a volumetric assessment of the fluid collections including every slice of the CT could provide better results than the present measurement on one CT slice. Yet, it was shown that the single slice measurement has a sufficient interreader agreement.^33^ Fifth, there was no adjustment for clinical features, such as age and immunosuppressive drugs, which were statistically significant between the fungal and non-fungal fluid collection group. There might be some bias as no multivariable analysis was performed.

In conclusion, CT texture features could be different between fungal infected and non-fungal infected fluid collections. The diagnostic overlap is large, which could reduce the clinical benefit. Further studies are needed to elucidate the possible diagnostic purpose of CT texture analysis for fluid collections.

## Data Availability

The analyzed anonymous study results can be provided from the corresponding author upon reasonable request.
